# The Lack of Sex, Age, and Anthropometric Diversity in Neck Biomechanical Data

**DOI:** 10.3389/fbioe.2021.684217

**Published:** 2021-08-17

**Authors:** Gabrielle R. Booth, Peter A. Cripton, Gunter P. Siegmund

**Affiliations:** ^1^ Orthopaedic and Injury Biomechanics Laboratory, School of Biomedical Engineering and Departments of Orthopaedics and Mechanical Engineering, University of British Columbia, Vancouver, BC, Canada; ^2^ International Collaboration on Repair Discoveries, University of British Columbia, Vancouver, BC, Canada; ^3^ MEA Forensic Engineers & Scientists, Richmond, BC, Canada; ^4^ School of Kinesiology, University of British Columbia, Vancouver, BC, Canada

**Keywords:** injury biomechanics, injury prevention, population diversity, neck, ageing, obesity, sex differences, anthropometric differences

## Abstract

Female, elderly, and obese individuals are at greater risk than male, young, and non-obese individuals for neck injury in otherwise equivalent automotive collisions. The development of effective safety technologies to protect all occupants requires high quality data from a range of biomechanical test subjects representative of the population at risk. Here we sought to quantify the demographic characteristics of the volunteers and post-mortem human subjects (PMHSs) used to create the available biomechanical data for the human neck during automotive impacts. A systematic literature and database search was conducted to identify kinematic data that could be used to characterize the neck response to inertial loading or direct head/body impacts. We compiled the sex, age, height, weight, and body mass index (BMI) for 999 volunteers and 110 PMHSs exposed to 5,431 impacts extracted from 63 published studies and three databases, and then compared the distributions of these parameters to reference data drawn from the neck-injured, fatally-injured, and general populations. We found that the neck biomechanical data were biased toward males, the volunteer data were younger, and the PMHS data were older than the reference populations. Other smaller biases were also noted, particularly within female distributions, in the height, weight, and BMI distributions relative to the neck-injured populations. It is vital to increase the diversity of volunteer and cadaveric test subjects in future studies in order to fill the gaps in the current neck biomechanical data. This increased diversity will provide critical data to address existing inequities in automotive and other safety technologies.

## Introduction

Injuries to the head and neck, which house and protect the brain and upper spinal cord, are some of the most catastrophic consequences of motor vehicle collisions. Over the past 7 decades, improvements in roads, vehicles, safety equipment, safety regulations, and enforcement have significantly reduced the injury, morbidity, and mortality burden associated with head and neck injuries. Despite these considerable achievements, many injury prevention approaches, including the computational models and anthropometric test devices (ATDs) used to design and evaluate safety equipment, have focused on 50th percentile adult male occupants (Linder and Svensson, 2019). As a result, females and others who fall outside the anthropometric envelope of this “median male” are not as well represented in automotive safety equipment design. Assuming that ATDs and other surrogates are appropriate for developing vehicle safety technology, it follows that considerable numbers of injured and killed occupants were using safety equipment that may not have been optimally designed for them.

European and United States traffic safety regulatory standards are used worldwide, with minor alterations, to assess a vehicle’s ability to prevent serious injuries for the occupants and other road users. These standards specify the use of 50th percentile adult male ATDs and representations of the 95th percentile male and 5th percentile female, which have been scaled from the 50th male by weight and height. While these three occupant representations attempt to approximate the median and extremes of height and weight of adult occupants, changes in size alone are not sufficient to represent the age, sex, and anthropometric variations seen in the population that safety technologies aim to protect (Linder and Svensson, 2019). Additionally, child restraint system performance evaluation is conducted using Hybrid III child dummies that were also derived from scalings of the Hybrid III 50th percentile adult male dummy and basic child anthropometry (Irwin and Mertz, 1997). Therefore, many ATDs used in traffic safety regulatory standards have been derived from a representation of a ‘median-sized’ adult male.

Anthropometry and size are not the only factors related to the use of ATDs that are median-male based. The injury assessment reference values (IARVs) for the neck, which were developed for relating the loads measured by ATDs to the potential for injury in humans, are based on a limited set of male human volunteer and cadaveric data (Mertz et al., 2003; Foster et al., 1977). These data have then been scaled using size (neck circumference) and tissue properties (calcaneal tendon strength) to provide IARVs for the 5th percentile female and 95th percentile male Hybrid III dummies. In the case of children, porcine models have been used to generate injury data, although the translation between animal and human data is outside the scope of the current study (Mertz et al., 2003).

There are considerable field data showing that female, elderly, and obese individuals are at greater risk than 50th percentile adult males of serious and fatal injuries across all body regions in similar severity collisions (Evans and Gerrish, 2001; Bédard et al., 2002; Hill and Boyle, 2006; Zhu et al., 2006; Bose, 2011; Rupp et al., 2013; Carter et al., 2014). Using data from the National Automotive Sampling System’s Crashworthiness Data System (NASS-CDS), Bose et al. (2011) found that the odds of a belt-restrained female driver sustaining severe injuries were 47% higher than those for a belt-restrained male driver involved in a comparable crash. Hill and Boyle (2006) found that females and older occupants (75+ year olds) were at a significantly higher risk of a severe injury in crashes recorded in the United States General Estimate System (GES). Evans and Gerrish (2001) used the Fatality Analysis Reporting System (FARS) database to compare the risk of fatal injury in two-car crashes where the sex of one driver was male and the other was female and found the fatality risk to be 22% greater for female drivers. Bédard et al. (2002) also used the FARS database to examine fatality risk in single vehicle collisions with fixed objects and found the odds ratio of fatal injury increases with age (OR = 4.98 for 80+ year olds) and female sex (OR = 1.54). Rupp et al. (2013) found that obese subjects were at an increased risk of AIS 3+ spine injuries. While other vehicle- and crash-related factors that co-vary with sex, age, and occupant obesity may explain some of these findings, biomechanical factors related to these variables are plausible explanations for some proportion of the observed effects.

A similar pattern of increased injury risk for female, elderly, and obese individuals is also observed specifically for head and neck injuries. Carter et al. (2014) found that older individuals have a greater risk of severe spine injuries in frontal and rollover crashes. Furthermore, females are at about double the risk of males for whiplash injuries in low speed rear-end collisions (Krafft et al., 2003; Jakobsson et al., 2004; Linder and Svensson, 2019). Moreover, active head restraints have been shown to be more effective for men than women (Kullgren et al., 2013).

Sex and anthropometry also affect the kinematics of individuals in collisions. In volunteer studies, females exhibit higher magnitude head accelerations in both frontal and rear-end collisions (Siegmund et al., 1997; van den Kroonenberg et al., 1998). In rear impacts, females also exhibit greater forward rebound and larger intersegmental motion between adjacent vertebrae in the cervical spine (Ono et al., 2006). Reed et al. (2012) studied obese and non-obese subjects and showed that excess slack was introduced in the belt system in obese subjects. In post-mortem human subjects (PMHSs), obese subjects experienced greater excursion and tended to pitch forward less than the non-obese subjects in 48 km/h frontal collisions (Kent et al., 2010). Computational models have also predicted higher neck displacements for females than for males in low-speed rear-end collisions (Viano, 2003) and poor concordance has been observed between the Global Human Body Models Consortium (GHBMC) finite element model and obese PMHS tests (Gepner et al., 2018).

Sex differences in external neck morphology and anatomical differences in the cervical spine have also been established. The vertebral anatomy, curvature, head mass, neck strength, neck muscle morphometry, and neck muscle activation patterns have all been shown to differ between males and females (Brault et al., 1998; Kamibayashi and Richmond, 1998; Matsumoto et al., 1998; Siegmund et al., 2003a; Klinich et al., 2004; Stemper et al., 2008; Vasavada et al., 2008; Sato et al., 2017).

The above review suggests that injury prevention technologies (e.g., restraint systems, airbags, and head restraints) have been primarily designed using representations and scalings of mid-sized male occupants, and that sex, age, and anthropometry potentially affect neck injury risk, head and neck kinematics, and ultimately neck injury mechanics in automotive collisions. While the biomechanics of these relationships and the degree to which sex, age, and anthropometry explain these relationships remains unclear, an important first step in addressing this potential inequity in injury prevention is to understand the diversity—or lack of diversity—in the baseline biomechanical data that inform our understanding of occupant kinematics and tolerances, and motivate our designs of ATDs and safety technologies. Therefore, our objective here is to quantify the distributions of sex, age, height, weight, and BMI for volunteer and PMHS tests that make up the available neck biomechanical data and to compare the distributions of these parameters to reference data drawn from the neck-injured, fatally-injured, and general populations.

## Methods

### Literature Search

A systematic search was performed for published studies that contained kinematic data for the head and torso in response to inertial loading and direct head and body impacts, and from which the neck response could be estimated. Five databases (PubMed, Web of Science Core Collection, Compendex Engineering Village, SportDiscus, and SAE Mobilus) were searched in June/July 2020 with no restrictions on year or language of publication. The search terms reflected the eligibility criteria, including keywords targeting human subjects and cadavers, head, neck and torso kinematics, and impact loading. Studies extracted from relevant review articles were also added to the results of these searches.

A sample Web of Science search is as follows:

#1 TS = (Volunteer* OR “*In Vivo*” OR Cadaver* OR “*Ex Vivo*” OR “Post mortem” OR PMHS).

#2 TS = (head).

#3 TS = (sled OR “crash test*" OR impact*)

#4 TS = (acceleration* OR displacement*)

#4 AND #3 AND #2 AND #1.

Studies from the search results were first compiled and deduplicated using Legacy RefWorks (ProQuest, Ann Arbor, MI). One author screened the titles and abstracts based on preset criteria (Table 1) and then performed a full-text review on the relevant subset to identify eligible studies containing the desired data using Covidence (Melbourne, Australia). A second author reviewed studies whose inclusion/exclusion was ambiguous. For eligible studies, we then determined if the kinematic data were available in the publication, appendix, supplementary material, by contacting the authors, or searching biomechanics databases (e.g., National Biodynamics Laboratory, Air Force Biodynamic, and NHTSA Biomechanics databases). We then extracted the sex, age, height, weight, and BMI for all volunteers and cadavers from each test within the included studies. These characteristics were compared to reference data for automotive neck injuries (NASS-CDS), automotive fatalities (FARS), and the general population (US Census Bureau, USCB).

**TABLE 1:** Study eligibility criteria.

Inclusion criteria	Exclusion criteria
• Test volunteer or cadaver subjects with or without helmets	• Solely use subjects who have undergone spinal surgery, have apparent or induced injuries, have been otherwise altered, or exhibit extreme spine pathologies
• Measure primary data on time history accelerations or displacements of both the human head and base of the neck or upper thorax (C6–T4 range)	• Involve modifying the kinematics of the head and neck through additional impacts (airbags, steering wheels, head restraints) or other factors
• Involve accelerating the head by means of inertial loading or direct head or body impact	• Poor methodology or insufficient detail to assess the quality of the methods used to obtain and modify data (requires the agreement of two reviewers)

### Reference Data

From the NASS-CDS dataset that had AIS codes (1993–2015), we extracted all cases with cervical spine injuries (Region 6, Structures 02, 50 and 59 based on the 1998 Abbreviated Injury Scale) for light vehicles (Body types 1–49) and all types of crashes. For each unique individual (*n* = 25,889), we extracted the maximum Abbreviated Injury Scale (AIS) score for their cervical spine injury, as well as their sex, age, height, weight, and BMI when present. Individuals were removed from the dataset if their sex was unknown (*n* = 9) or if their age, height, and weight were all unreported (*n* = 4). Individuals with BMI >76 were removed, as there were continuous data up to a BMI of 76, after which the values doubled and were assumed to be errors (*n* = 17). The data were then grouped into three datasets based on injury severity: AIS1+, AIS2+, and AIS3+ injuries. Injuries of unknown severity (coded as AIS 7 in NASS) were included in the AIS1+ group but removed from AIS2+ and AIS3+ groups. Pregnant females were included in the age and height datasets but excluded from the weight and BMI datasets.

From the FARS data, we queried the Fatality and Injury Reporting System Tool (FIRST) to extract the sex and age of all drivers and occupants who died in motor vehicle crashes in the full date range of the available data (2005–2019). The FARS database did not contain height or weight data. The FARS data included deaths from all types of injuries, not just cervical spine injuries.

From the census data, we extracted the estimated 2017 United States population for females and males at each year of age between 0 and 100 years (US Census Bureau, 2021). All individuals over 100 years old were pooled into the 100-years category. To estimate the height and weight distributions of the general population, we first fit a lognormal distribution to the percentile distribution data (5, 10, 15, 25, 50, 75, 85, 90, and 95th percentiles) of the height and weight data for each sex and year of age (Fryar et al., 2021) and then calculated a weighted sum of these distributions based on the number of people in each age group. Separate height and weight distributions were used for each year from 2 to 19 years and for each decade thereafter (e.g., 20–29 years, 30–39 years, ... , 70–79 years, 80+ years). No information on the correlation between height and weight was available, therefore BMI for the general population was not computed.

### Data Distributions

Histograms for age, height, weight and BMI were created for the number of volunteer tests, PMHS tests, AIS1+ injured individuals, AIS2+ injured individuals, AIS3+ injured individuals, fatalities, and people in the general population. We focused our analysis on the number of volunteer and PMHS tests rather than the number of volunteers or cadavers because each test yielded a unique set of data. As a result, a volunteer or cadaver could appear multiple times in the histograms. The histograms pooled both sexes and used bin widths of 1 year, 1 cm, 1 kg, and 1 kg/m^2^ for age, height, weight, and BMI, respectively. For the AIS data, the bin widths for height were set to 2.54 cm (1 inch) and 2.258 kg (5 pounds) for weight. Separate density distributions for each sex were then generated using kernel density estimates (geom_density function in R). For a dataset with N observations, this function yields the sum of *i* = 1 to N normal distributions, where the mean of the *i*th distribution equals the value of the *i*th observation and the standard deviation for all N distributions equals the optimum bandwidth (Silverman, 1986). The optimum bandwidth for each dataset was calculated using Eq. 1 (Silverman, 1986, pg 48), and then all bandwidths for a given parameter (age, height, weight, or BMI) were averaged to select a common bandwidth for all distributions of the same parameter. The average bandwidths for each parameter were as follows: age 2.90 years, height 1.40 cm, weight 2.95 kg, and BMI 1.01 kg/m^2^. The bandwidth for height was doubled from 0.70 to 1.40 cm as the average optimum bandwidth created unrealistic peaks in the data with 1-cm bin widths.

$$\text{Optimum}\;\text{bandwidth}=0.9\min\left(\text{SD},\text{IQR}/1.34\right)\times {\text{N}}^{-0.2}$$ (1)

where SD = standard deviation of the dataset, and IQR = interquartile range of the dataset.

The histograms related to all distributions for a single parameter were plotted at the same scale, i.e., the areas of all related histograms are equal to one. The relative areas under the female and male distributions reflect their relative proportions of the population. The areas for the female and male distributions were doubled, i.e., their sum is double the area of the histogram, to improve their visibility relative to the histograms. The medians for the male and female data were computed using all of the data within a dataset. For the general population, the medians for the height and weight of adults (≥16 years) were also calculated. Dispersion within each of the datasets was quantified using the interquartile range (IQR).

## Results

**FIGURE 1:**
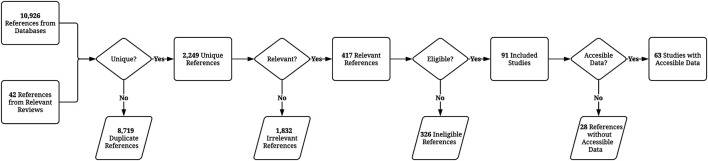
Flowchart showing the number of studies at each stage of the selection process.

**FIGURE 2:**
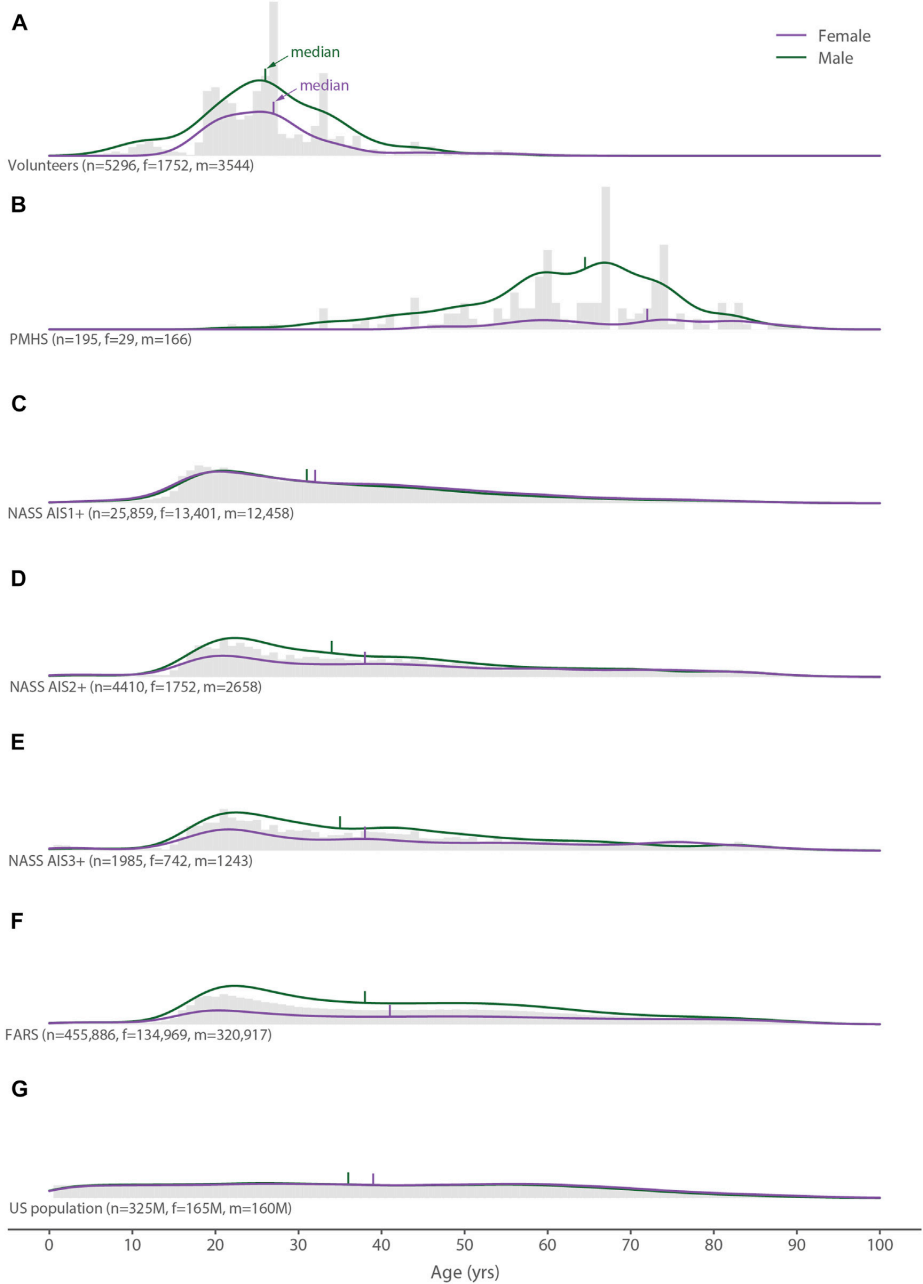
Distribution of all age data (gray histograms), females (purple lines), and males (green lines) for **(A)** the volunteer tests, **(B)** the PMHS tests, **(C)** the NASS AIS1+ data, **(D)** the NASS AIS2+ data, **(E)** the NASS AIS3+ data, **(F)** the FARS data, and **(G)** the United States population.

**FIGURE 3:**
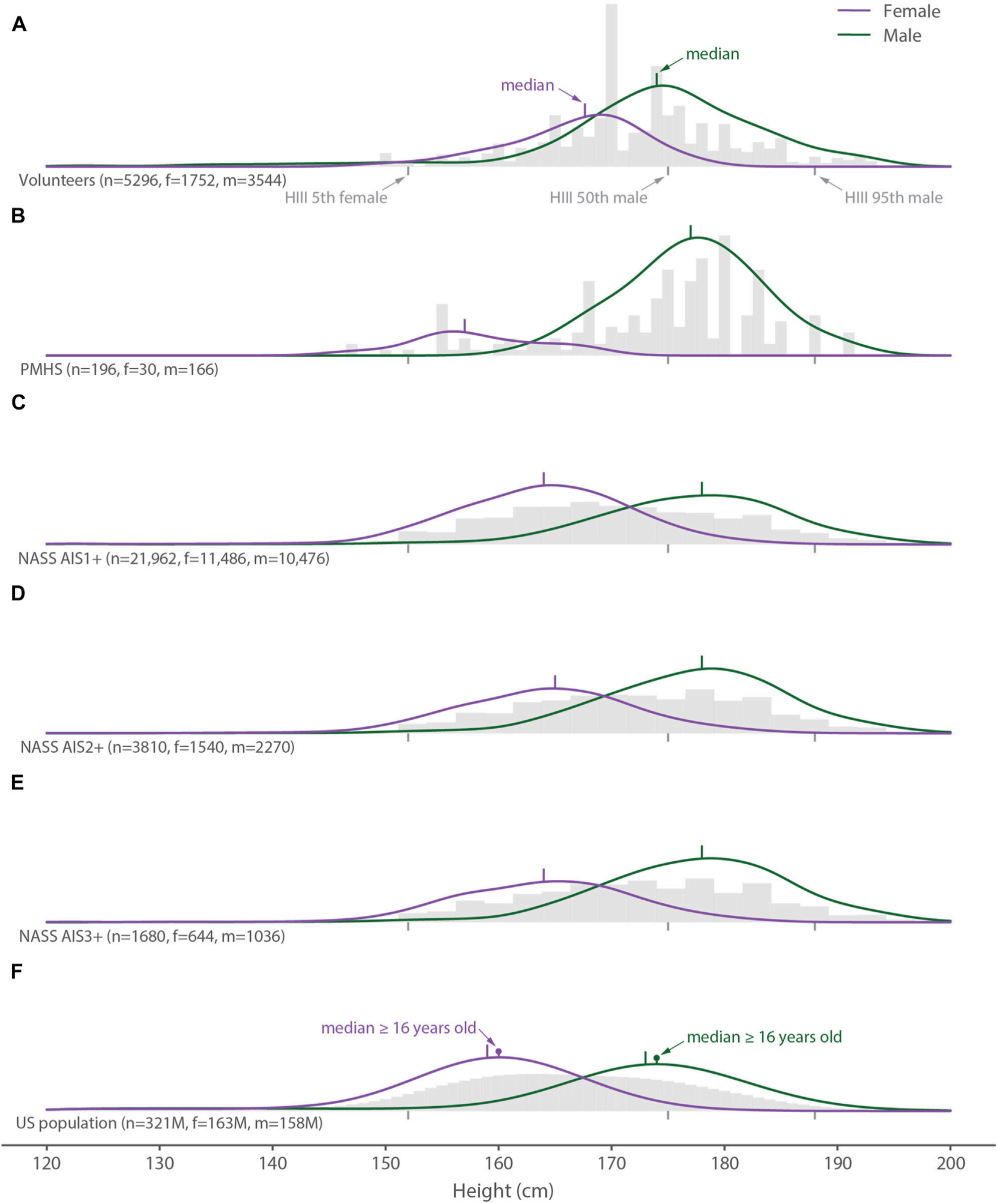
Distribution of all height data (gray histograms), females (purple lines) and males (green lines) for **(A)** the volunteer tests, **(B)** the PMHS tests, **(C)** the NASS AIS1+ data, **(D)** the NASS AIS2+ data, **(E)** the NASS AIS3+ data, and **(F)** the United States population. The gray vertical bars below each histogram show the heights of the 5th percentile female, 50th percentile male, and 95th percentile male Hybrid III crash test dummies.

**FIGURE 4:**
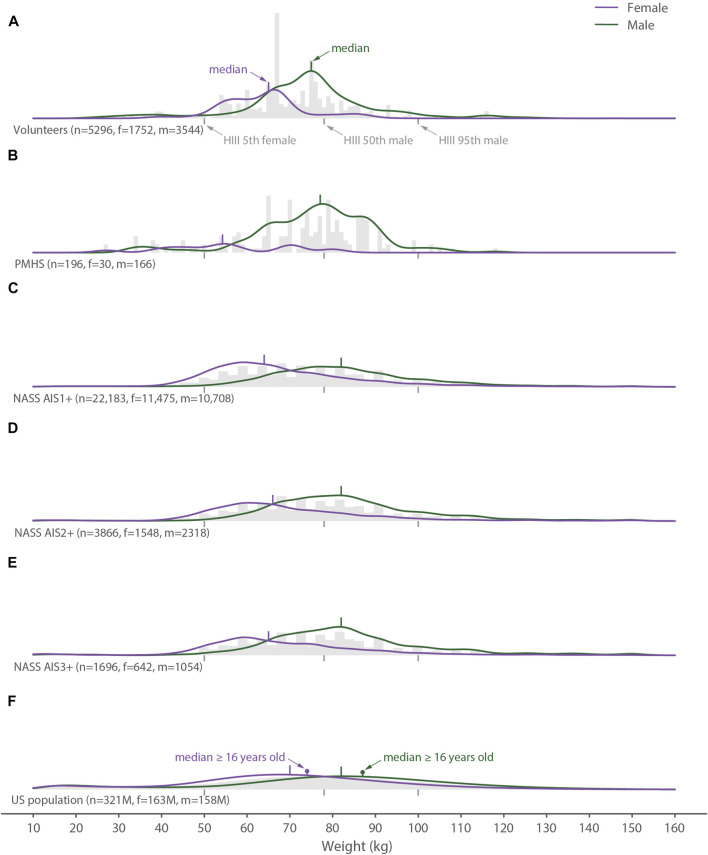
Distribution of all weight data (gray histograms), females (purple lines) and males (green lines) for **(A)** the volunteer tests, **(B)** the PMHS tests, **(C)** the NASS AIS1+ data, **(D)** the NASS AIS2+ data, **(E)** the NASS AIS3+ data, and **(F)** the United States population. The gray vertical bars below each histogram show the weights of the 5th percentile female, 50th percentile male, and 95th percentile male Hybrid III crash test dummies.

**FIGURE 5:**
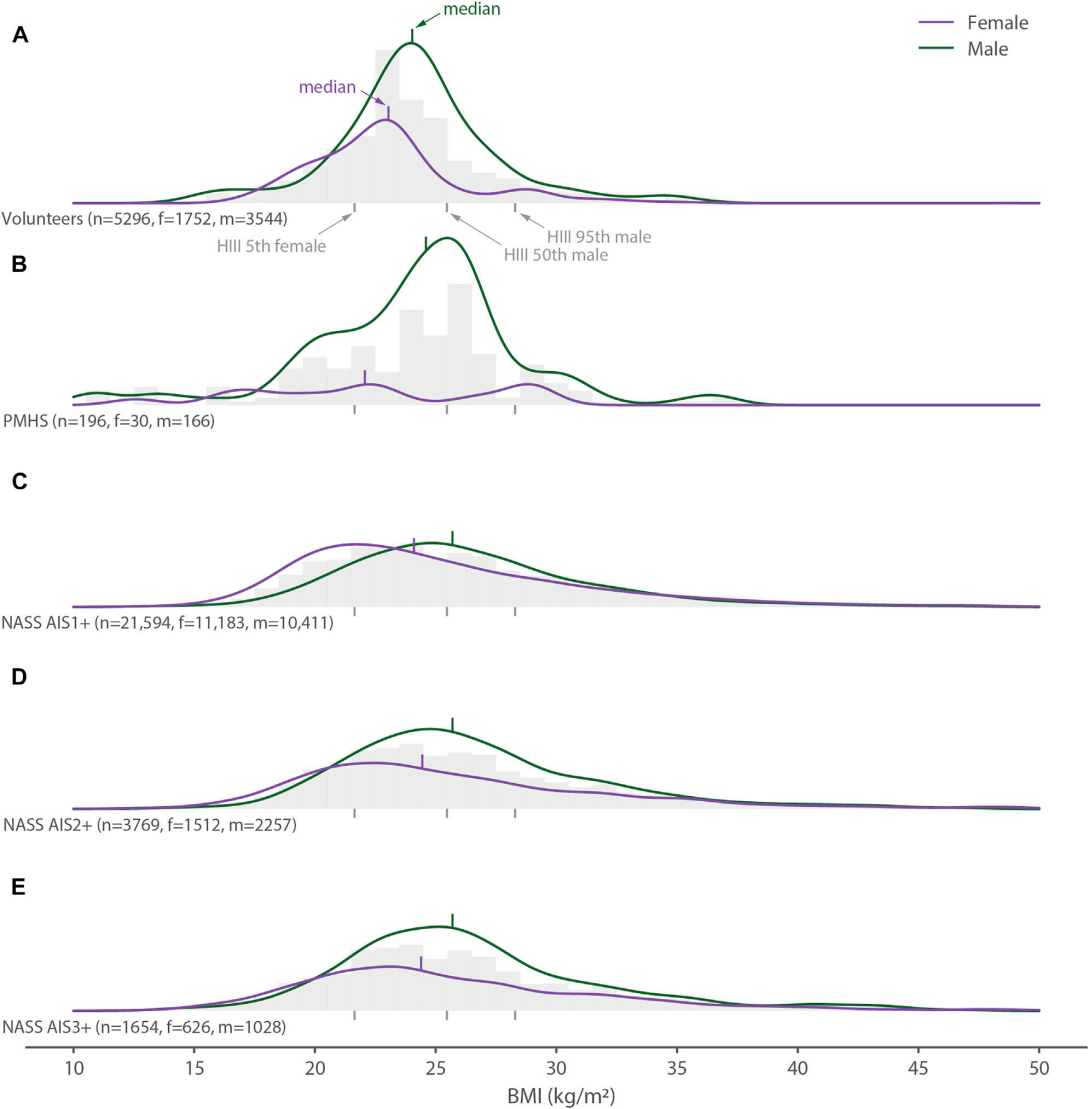
Distribution of all body mass index (BMI) data (gray histograms), females (purple lines) and males (green lines) for **(A)** the volunteer tests, **(B)** the PMHS tests, **(C)** the NASS AIS1+ data, **(D)** the NASS AIS2+ data, and **(E)** the NASS AIS3+ data. The gray vertical bars below each histogram show the heights of the 5th percentile female, 50th percentile male and 95th percentile male Hybrid III crash test dummies.

Our search yielded 2,249 unique studies, of which 417 studies were relevant to our objectives, 91 of the 417 relevant studies measured the kinematic variables we sought, and 63 of these studies presented or otherwise allowed access to their data (Figure 1). The 63 studies contained 999 unique volunteers exposed to 5,229 tests and 110 unique PMHSs exposed to 202 tests (Ewing et al., 1969; Ewing and Thomas, 1972; Ewing et al., 1975; Ewing et al., 1977, Ewing et al., 1978; Kallieris et al., 1987; Buhrman and Perry, 1994; Margulies et al., 1998; Morris and Popper, 1999; Ono et al., 1999; Yoganandan and Pintar, 2000; Davidsson et al., 2001; Meijer et al., 2001; Fugger et al., 2002; Petitjean et al., 2002; Vezin et al., 2002; Deng and Wang, 2003; Perry et al., 2003; Siegmund et al., 2003a; Siegmund et al., 2003b; Vezin and Verriest, 2003; Doczy et al., 2004; Siegmund et al., 2004; Blouin et al., 2006; Rouhana et al., 2006; Wiechel and Bolte, 2006; Ejima et al., 2007; Pintar et al., 2007; Ejima et al., 2008; Siegmund et al., 2008; Arbogast et al., 2009; Funk et al., 2009; Lopez-Valdes et al., 2009; Siegmund and Blouin, 2009; White et al., 2009; Lopez-Valdes et al., 2010; Pintar et al., 2010; Funk et al., 2011; Sundararajan et al., 2011; Arbogast et al., 2012; Ejima et al., 2012; Stammen et al., 2012; Symeonidis et al., 2012; Forman et al., 2013; Mathews et al., 2013; Poulard et al., 2013; van Rooij et al., 2013; Crandall et al., 2014; Gutsche et al., 2014; Lessley et al., 2014; Lopez-Valdes et al., 2014; Seacrist et al., 2014; Shaw et al., 2014; Acosta et al., 2016; López-Valdés et al., 2016; Pietsch et al., 2016; Albert, Beeman and Kemper, 2018; Holt et al., 2018; Humm et al., 2018; Petit et al., 2019; Stark et al., 2019; Zaseck et al., 2019; Holt et al., 2020). About 66% of the volunteer tests and 84% of the PMHS tests were conducted with males (Table 2, also visible in Figures 2–5). Both values were higher than the proportion of males in the United States population (49%) and in the AIS1+, AIS12+, and AIS13+ neck injury groups (48, 60, and 63%, respectively), but landed on either side of the proportion of males seen in United States automotive fatalities (70%).

**TABLE 2:** Sample size (n), median, and interquartile range (IQR) of the age, height, weight, and BMI data for the volunteer tests, PMHS tests, AIS1+, AIS2+, and AIS3+ from the NASS data, FARS data, and the United States population.

		Age (years)			Height (cm)			Weight (kg)			BMI (kg/m^2^)		
		n	Median	IQR	n	Median	IQR	n	Median	IQR	n	Median	IQR
Volunteers	Total	5,296	26	11	5,296	172	10	5,296	69	14	5,296	23	3
	Male	3,544	26	11	3,544	174	10	3,544	75	13	3,544	24	3
	Female	1752	27	7	1752	168	6	1752	65	10	1752	23	2
PMHS	Total	195	65	17	196	176	11	196	75	17	196	24	5
	Male	166	65	15	166	177	6	166	77	18	166	25	4
	Female	29	72	20	30	157	7	30	54	25	30	22	9
AIS1+	Total	25,859	31	24	21,962	170	15	22,183	73	25	21,594	25	7
	Male	12,458	31	23	10,476	178	10	10,708	82	21	10,411	26	6
	Female	13,401	32	25	11,486	165	10	11,475	64	20	11,183	24	8
AIS2+	Total	4,410	35	29	3,810	173	15	3,866	76	24	3,769	25	7
	Male	2,658	34	26	2,270	178	10	2,318	82	19	2,257	26	6
	Female	1752	38	35	1,540	165	12	1,548	66	22	1,512	24	8
AIS3+	Total	1985	36	30	1,680	173	15	1,696	77	24	1,654	25	7
	Male	1,243	35	26	1,036	178	10	1,054	82	19	1,028	26	6
	Female	742	38	36	644	164	13	642	65	20	626	24	8
FARS	Total	455,886	39	33	—	—	—	—	—	—	—	—	—
	Male	320,917	38	31	—	—	—	—	—	—	—	—	—
	Female	134,969	41	38	—	—	—	—	—	—	—	—	—
United States Pop	Total	324,982,000	38	38	321,006,000	165	17	321,013,000	75	33	—	—	—
	Male	160,044,000	36	37	158,034,000	173	12	158,035,000	82	33	—	—	—
	Female	164,938,000	39	38	162,972,000	159	11	162,978,000	70	30	—	—	—

Of the four variables we examined, age showed the largest differences between datasets (Figure 2). The median ages for the PMHS tests were higher than all of the reference datasets, ranging from as little as 26 years older than the FARS data (males) up to 40 years older than the AIS1+ data (females). The youngest female and male PMHSs tested were 46 and 22 years old, respectively. The median ages for the volunteer tests, on the other hand, were lower than all of the reference datasets by a maximum of 14 years relative to the FARS data (females). In addition to differences in the medians, the age-related dispersions (IQRs) of both the volunteer tests and the PMHS tests were less than all of the reference datasets (Table 2). Dispersion was smallest for the female volunteer tests (7 years) and largest for the female fatalities and female population data (38 years). There were no volunteer or PMHS tests for female children or adolescents (≤17 years old) and the oldest female and male volunteer test subjects were 63 and 65 years old, respectively.

The median height of the female PMHS tests was only 2 cm shorter than the median female in the United States population, but 6–7 cm shorter than females with neck injuries (Figure 3). In contrast, the median height of the female volunteer tests was 3–4 cm taller than the females with neck injuries. For males, the median height for the volunteer tests was the same as the median for the adult United States population (Figure 3), but 3–4 cm shorter than the median heights for the PMHS tests and the neck injured populations. The dispersions in height for the female volunteer data and for both the male and female PMHS data were less than the dispersion for the neck-injured population and the general population.

The weight data exhibited a similar pattern to the height data. The median weight of the female PMHS tests was 10–12 kg less than the females in the neck-injured groups and 16 kg less than females in the general population (Figure 4). For males, the median weights for the volunteer and PMHS tests were 5–7 kg less than both the neck-injured and general populations. Dispersion in the weight of the female volunteer tests was about half of the neck injured population and a third of the general population, whereas the dispersion in the PMHS data fell within the range between the neck-injured and general populations. For males, the dispersion in the volunteer tests was also about one third of the general population, but the PMHS and neck-injured populations were similar to one another.

Volunteers and PMHSs had slightly lower median BMIs than seen in the neck-injured populations (Figure 5). The median BMIs of the male volunteers and the female PMHSs differed the most, by 2 kg/m^2^, from the neck-injury populations. Dispersion in the volunteer BMI’s was one half of the neck-injured population for females and one quarter of the neck-injured population in males.

## Discussion

Our goal was to quantify the sex, age, and anthropometry of the volunteers and cadavers that comprise the available kinematic data for the human neck and to compare the distributions of these variables to those of the neck-injured, fatally-injured, and general populations. Overall, we found large differences in the sex and age distributions between the biomechanical data and the reference populations, and smaller, primarily female-specific, differences in the height, weight, and BMI distributions between the biomechanical data and reference populations. These findings point to an underlying lack of diversity in the biomechanical data being used to understand and ultimately prevent collision-related neck injuries.

The most obvious difference between the biomechanical and reference datasets is between males and females. There were twice as many male volunteer tests as female volunteer tests (67% male vs. 33% female) and over five times as many male PMHS tests as female PMHS tests (85 vs. 15%). In contrast, there were fewer males than females (48 vs. 52%) with neck injuries across the entire range of severities (i.e., AIS1+) and 1.67 times more males than females (63 vs. 37%) when only serious and more severe neck injuries (AIS3+) were considered. Although males were ∼2.4 times more likely than females (70 vs. 30%) to die in a road crash based on FARS data, this database captures deaths from non-neck-related trauma and is therefore a poorer reference for the appropriate diversity needed in the neck biomechanical data. Based on these findings, more biomechanical data are needed for females throughout the neck injury spectrum—from whiplash injury to neck fractures—although the optimum sex distribution of volunteers and PMHSs may vary for different neck injuries. For instance, volunteer tests may be more relevant for studying AIS1 injuries and therefore a bias toward more female than male subjects should be considered to better reflect the AIS1 injured population; whereas cadaver tests may be more relevant for studying severe neck injuries and therefore a bias toward more male than female cadavers—albeit less bias than currently exists—could be considered to reflect the AIS3+ injured population. More generally, our findings suggest that the applicability of biomechanical research could be improved if researchers queried the available field data for sex and anthropometry distributions relevant to the injury of interest and then enrolled volunteers and/or cadavers to match.

The age differences we observed between the biomechanical and reference datasets were primarily in the PMHS data. This finding is not surprising given that 75% of deaths in males and 85% of deaths in females occur at over 65 years of age (Shumanty, 2018), making old cadavers more readily available to researchers. Nevertheless, there are established age-related changes in tissue morphology and failure response that potentially confound comparisons between the volunteer and PMHS data (Yukawa et al., 2012; Yoganandan et al., 2018). These differences create problems when combining the geometric, kinematic, and neuromuscular data of young volunteers with the failure data of old cadavers, particularly when creating human body models, developing injury assessment reference values, or designing safety interventions. For example, the neck IARVs developed for correlating injury tolerances with the Hybrid III are scaled using tissue properties which are likely to be biased by the older age of cadaveric specimens (Mertz et al., 2003). Another key age-related difference is the complete absence of volunteer and cadaveric data for female children and adolescents. While injury rates to this sub-population are relatively low, the societal costs of injury to children are high and therefore biomechanical data from both sexes are needed to first understand if differences exist and then how to accommodate for them if present.

Though not a goal of the study, we observed that the neck-injured population was taller than the general population. The reasons for this difference cannot be discerned directly from our data, but possible explanations include a longer distance between the inertial mass of the head and the fulcrum created by the shoulder belt crossing the chest and shoulder during frontal crashes, a greater chance of head contact and neck loading during other types of impacts, and different interactions with airbags. Further work is needed to better understand this pattern and its possible importance when recruiting volunteers and selecting cadavers for studying neck injury. The height of female cadavers was even shorter than the general population and therefore matched the distribution of neck-injured females even more poorly. The heights of the three common Hybrid III dummies (5th female, 50th male, and 95th male; shown in Figure 3) appear to cover the range of injured individuals but result in many of the females landing in the gap between the 5th female and the 50th male dummy. Moreover, a median height for the neck-injured male population that is ∼5 cm taller than the 50th-percentile male dummy, which is the most commonly used dummy for vehicle standards testing, may not be optimizing vehicle safety for taller male occupants.

In contrast to height, the weight distributions of neck-injured individuals and both the volunteer and PMHS data are lower than for the general population. The weight of female PMHSs is low compared to the other distributions, possibly signifying attempts by researchers to generate data related to the 5th-percentile female dummy. Although the BMI distribution of the general population was not determined because the covariance of height and weight was not available, the volunteer and PMHS test data was below the median levels for the neck-injured populations.

To interpret our findings, one should consider the different kinds of biomechanical data generated from volunteer and PMHS tests. Volunteers are exposed to lower, often sub-injurious conditions and the acquired data consist of kinematics from external sensors or motion tracking, intervertebral kinematics acquired via fluoroscopy, muscle activation data from surface or in-dwelling electromyography, kinetics computed via inverse dynamics, and potentially subjective or objective clinical data (including pre- and post-test imaging). Volunteer data can yield information related to realistic initial postures, neuromuscular responses, and potential pain measures. Cadavers, on the other hand, are often exposed to injurious loading conditions. These are the only human subjects that can be exposed to injurious or potentially injurious loads. The acquired data from PMHS tests consist of kinematic and kinetic data from external/embedded sensors or motion tracking, intervertebral data from high-speed x-ray, pre- and post-test imaging, and post-test dissection to identify injuries. Cadaver data can yield information regarding the tolerance to injuries detected via imaging, visible inspection and/or dissection, or post-impact mechanical testing. Given these differing conditions, outcomes, and ethical considerations, volunteer data may be more relevant to less severe neck injuries whereas PMHS data may be more relevant to more severe neck injuries.

Although our findings showed differences in the sex, age, and anthropometry of the biomechanical and reference populations, our analysis did not reveal whether the presence or scale of these differences was important. Previously documented morphological (Siegmund et al., 1997; Kamibayashi and Richmond, 1998; Matsumoto et al., 1998; Klinich et al., 2004; Stemper et al., 2008; Vasavada et al., 2008; Sato et al., 2017) and physiological differences (Ono et al., 2006; Vasavada et al., 2008) between male and female necks combined with the different risks for spine injuries in males and females in frontal and rollover crashes (Carter et al., 2014) suggests that some sex or sex-related variables could be responsible, but our understanding of the complex relationships amongst the many potential variables remains incomplete. For instance, sex, height, and weight are all interrelated, and even “normalized” metrics like BMI vary with sex and other variables (Heymsfield et al., 2014), and one variable could act as a surrogate for another in an exploratory correlational analysis. More mechanistic approaches, where individual variables or a small number of variables are systematically explored, are needed to determine which variables are most important for a specific injury. Other factors, such as hormones, health, prior injury, disease state, and other variables further complicate our understanding of neck injury biomechanics.

We chose to tabulate volunteer and PMHS tests rather than the individual volunteers and cadavers. While we recognize that multiple tests from a single volunteer/cadaver do not generate independent data, many of the tests were not identical and therefore generated different, though not wholly independent data. From this perspective, our analysis provides an optimistic view of the amount of biomechanical data available for the human neck, and yet it still shows that there are large gaps in the overlap between the biomechanical data, the neck-injured population and the general population. A parallel set of figures reporting the data for individual volunteers and cadavers showed similar results (see the Supplementary Materials). In these alternate figures male subjects outnumber female subjects, the biases toward young volunteers and old cadavers remain, and the female anthropometry data remained shifted toward the 5th-percentile female.

The median and distribution of human anthropometry varies temporally and across the world’s regions (Lee and Bro, 2008), and therefore using a reference population from a single year and country provides a perspective that may not be relevant to another year or country. We used recent measures of the United States population as a reference to directly compare with the United States injury datasets, however any population of interest to future researchers could be compared with the volunteer and PMHS figures. Additionally, safety systems in automobiles have changed considerably since 1993 and may confound our injury curves. To explore the effect of the differing time periods on the injury data, we split the NASS dataset into two groups: data preceding (1993–2004) and data overlapping (2005–2015) the available FARS data period. The greatest differences between the two groups were for age and weight. If we were to plot only the data from the later group, then compared to the overall data shown in the figures the mean age would increase 2.0 years for AIS1+, 1.1 years for AIS2+ and 0.7 years for AIS3+, whereas the average weight would increase 2.2 kg for AIS1+ and AIS2+ and 2.0 kg for AIS3+. Thus, at maximum, the age and weight histograms presented in the figures would shift about one bin width to the right, but would not change our overall findings. Another limitation of our work is that we did not separate either the biomechanical data or the neck-injured population by loading direction, crash type or injury type. Nevertheless, we recommend that researchers planning to conduct future volunteer and cadaver tests consider these specific factors when they specify or set up recruitment plans for the sex, age, and anthropometry distributions of their volunteers and cadavers.

## Conclusion

We found large differences in the distributions of sex and age between the populations used to generate biomechanical data for the human neck and the neck-injured populations. Smaller differences were noted in the height, weight, and BMI distributions between these populations. Overall, our findings indicate that more female biomechanical data are needed, especially for females of average height and weight. Our findings also show that there is minimal biomechanical data for older volunteers, young cadavers, and volunteers of both sexes with high BMIs. More generally, we encourage researchers to consider the diversity of the population being injured when enrolling volunteers and cadavers for their biomechanical studies.
