# Supplementary material for: The Lack of Sex, Age, and Anthropometric Diversity in Neck Biomechanical Data
Source: Front Bioeng Biotechnol. 2021 Aug 17;9:684217. doi: 10.3389/fbioe.2021.684217 (PMC8416072; doi:10.3389/fbioe.2021.684217)
Supplement: Supplementary file 1 [file DataSheet1.PDF]

Supplemental Material

Table 1. The 63 included studies with accessible kinematic data.

Year	First Author	Study Title
2016	Acosta	Comparison of Whole Body Response in Oblique and Full Frontal Sled Tests
2018	Albert	Occupant kinematics of the Hybrid III, THOR-M, and postmortem human surrogates under various restraint conditions in full-scale frontal sled tests
2009	Arbogast	Comparison of Kinematic Responses of the Head and Spine for Children and Adults in Low-Speed Frontal Sled Tests
2012	Arbogast	The Effect of Pretensioning and Age on Torso Rollout in Restrained Human Volunteers in Far-Side Lateral and Oblique Loading
1994	Buhrman	Human and Mannequin Head Neck Response to +G(z) Acceleration when Encumbered by Helmets of various Weights
2006	Blouin	Auditory Startle Alters the Response of Human Subjects Exposed to a Single Whiplash-like Perturbation
2014	Crandall	Displacement Response of the Spine in Restrained PMHS During Frontal Impacts
2001	Davidsson	Human Volunteer Kinematics in Rear-End Sled Collisions
2003	Deng	Assessment of H-Model Using Volunteer Tests
2004	Doczy	The Effects of Variable Helmet Weight and Subject Bracing on Neck Loading During Frontal -Gx Impact
2012	Ejima	Effects of Pre‑impact Swerving/Steering on Physical Motion of the Volunteer in the Low‑Speed Side‑impact Sled Test
2007	Ejima	A study on occupant kinematics behaviour and muscle activities during pre-impact braking based on volunteer tests
2008	Ejima	Prediction of the physical motion of the human body based on muscle activity during pre-impact braking
1975	Ewing	Effect of the Initial Position of the Head and Neck on the Dynamic Response of the Human Head and Neck to -Gx Impact Acceleration
1978	Ewing	Effect of initial position on the human head and neck response to +Y impact acceleration
1977	Ewing	Dynamic response of the human head and neck to +Gy impact acceleration
1969	Ewing	Living Human Dynamic Response to —Gx Impact Acceleration II—Accelerations Measured on the Head and Neck
1972	Ewing	Human head and neck response to impact acceleration
2013	Forman	Occupant Kinematics and Shoulder Belt Retention in Far-Side Lateral and Oblique Collisions: A Parametric Study
2002	Fugger	Human Occupant Kinematics in Low Speed Side Impacts
2009	Funk	Validation and Application of a Methodology to Calculate Head Accelerations and Neck Loading in Soccer Ball Impacts
2011	Funk	Head and Neck Loading in Everyday and Vigorous Activities

2014	Gutsche	Comparison of the cervical spine bony kinematics for female PMHS with the virtual EvaRID dummy under whiplash loading.
2018	Holt	Effect of countermeasures on adult kinematics during pre-crash evasive swerving
2020	Holt	The effect of vehicle countermeasures and age on human volunteer kinematics during evasive swerving events
2018	Humm	Three-dimensional kinematic corridors of the head, spine, and pelvis for small female driver seat occupants in near- and far-side oblique frontal impacts
1987	Kallieris	Comparison of human volunteer and cadaver head-neck response in frontal flexion
2014	Lessley	Occupant Kinematics in Laboratory Rollover Tests: PMHS Response
2010a	López-Valdés	A comparison between a child-size PMHS and the Hybrid III 6 YO in a sled frontal impact
2010b	López-Valdés	Analysis of spinal motion and loads during frontal impacts. Comparison between PMHS and ATD
2014	López-Valdés	The Six Degrees of Freedom Motion of the Human Head, Spine, and Pelvis in a Frontal Impact
2016	López-Valdés	Analysis of occupant kinematics and dynamics in nearside oblique impacts
1998	Margulies	Kinematic Response of the Neck to Voluntary and Involuntary Flexion
2013	Mathews	Electromyography responses of pediatric and young adult volunteers in low-speed frontal impacts
2001	Meijer	Analysis of rear end impact response using mathematical human modelling and volunteer tests
1999	Morris	Gender and effect of impact acceleration on neck motion
1999	Ono	Relationship between Localized Spine Deformation and Cervical Vertebral Motions for Low Speed Rear Impacts Using Human Volunteers
2003	Perry	The Effects of Variable Helmet Weight on Head Response and Neck Loading During Lateral +Gy Impact
2019	Petit	Far Side Impact Injury Threshold Recommendations Based on 6 Paired WorldSID / Post Mortem Human Subjects Tests
2002	Petitjean	Laboratory Reconstructions of Real World Frontal Crash Configurations Using the Hybrid III and THOR Dummies and PMHS
2016	Pietsch	Evaluation of WIAMan Technology Demonstrator Biofidelity Relative to Sub-Injurious PMHS Response in Simulated Under-body Blast Events
2007	Pintar	Comparison of PMHS, WorldSID, and THOR-NT Responses in Simulated Far Side Impact
2010	Pintar	Lower Cervical Spine Loading in Frontal Sled Tests Using Inverse Dynamics: Potential Applications for Lower Neck Injury Criteria
2013	Poulard	In Vivo Analysis of Thoracic Mechanical Response Variability under Belt Loading: Specific Behavior and Relationship to Age, Gender and Body Mass Index
2006	Rouhana	Biomechanics of 4-Point Seat Belt Systems in Farside Impacts

2014	Seacrist	Evaluation of pediatric ATD biofidelity as compared to child volunteers in low-speed far-side oblique and lateral impacts
2014	Shaw	Side Impact PMHS Thoracic Response With Large-Volume Air Bag
2003A	Siegmund	Awareness affects the response of human subjects exposed to single whiplash perturbations
2003B	Siegmund	Rapid neck muscle adaptation alters the head kinematics of aware and unaware subjects undergoing multiple whiplash-like perturbation
2004	Siegmund	Gradation of Neck Muscle Responses and Head/Neck Kinematics to Acceleration and Speed Change in Rear-end Collisions
2008	Siegmund	Are cervical multifidus muscles active during whiplash and startle?<br>An initial experimental study
2009	Siegmund	Head and neck control varies with perturbation acceleration but not jerk: implications for whiplash injuries
2012	Stammen	Dynamic Properties of the Upper Thoracic Spine-Pectoral Girdle (UTS-PG) System and Corresponding Kinematics in PMHS Sled Tests
2019	Stark	Human Response and Injury Resulting from Head Impacts with Unmanned Aircraft Systems
2011	Sundararajan	Biomechanical Assessment of a Rear-Seat Inflatable Seatbelt in Frontal Impacts
2012	Symeonidis	Analysis of the stability of PTW riders in autonomous braking scenarios
2013	van Rooij	Volunteer Kinematics and Reaction in Lateral Emergency Maneuver Tests
2002	Vezin	Comparison of Hybrid III, Thor-α and PMHS Response in Frontal Sled Tests
2003	Vezin	Influence of the Impact and Restraint Conditions on Human Surrogate Head Response to a Frontal Deceleration
2009	White	Investigation of upper body and cervical spine kinematics of Post Mortem Human Subjects (PMHS) during low-speed, rear-end impacts
2006	Wiechel	Response of reclined post mortem human subjects to frontal impact
2000	Yoganandan	Biomechanics of human occupants in simulated rear crashes: documentation of neck injuries and comparison of injury criteria
2020	Zaseck	Kinematic and Biomechanical Response of Post-Mortem Human Subjects Under Various Pre-Impact Postures to High-Rate Vertical Loading Conditions

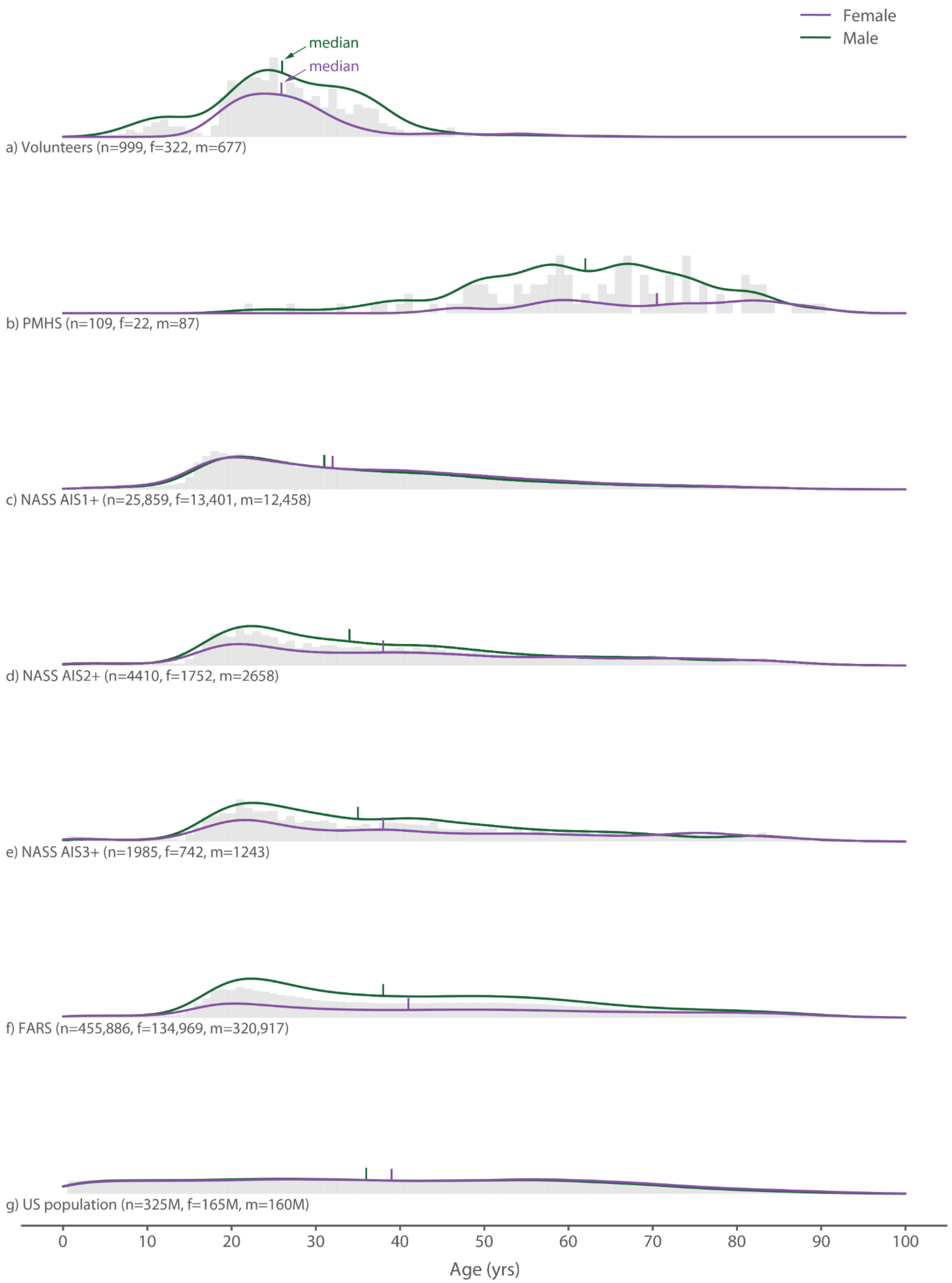

Figure 1. Distribution of all age data (gray histograms), females (purple lines), and males (green lines) for a) the unique volunteers, b) the unique PMHSs, c) the NASS AIS1+ data, d) the NASS AIS2+ data, e) the NASS AIS3+ data, f) the FARS data, and g) the US population.

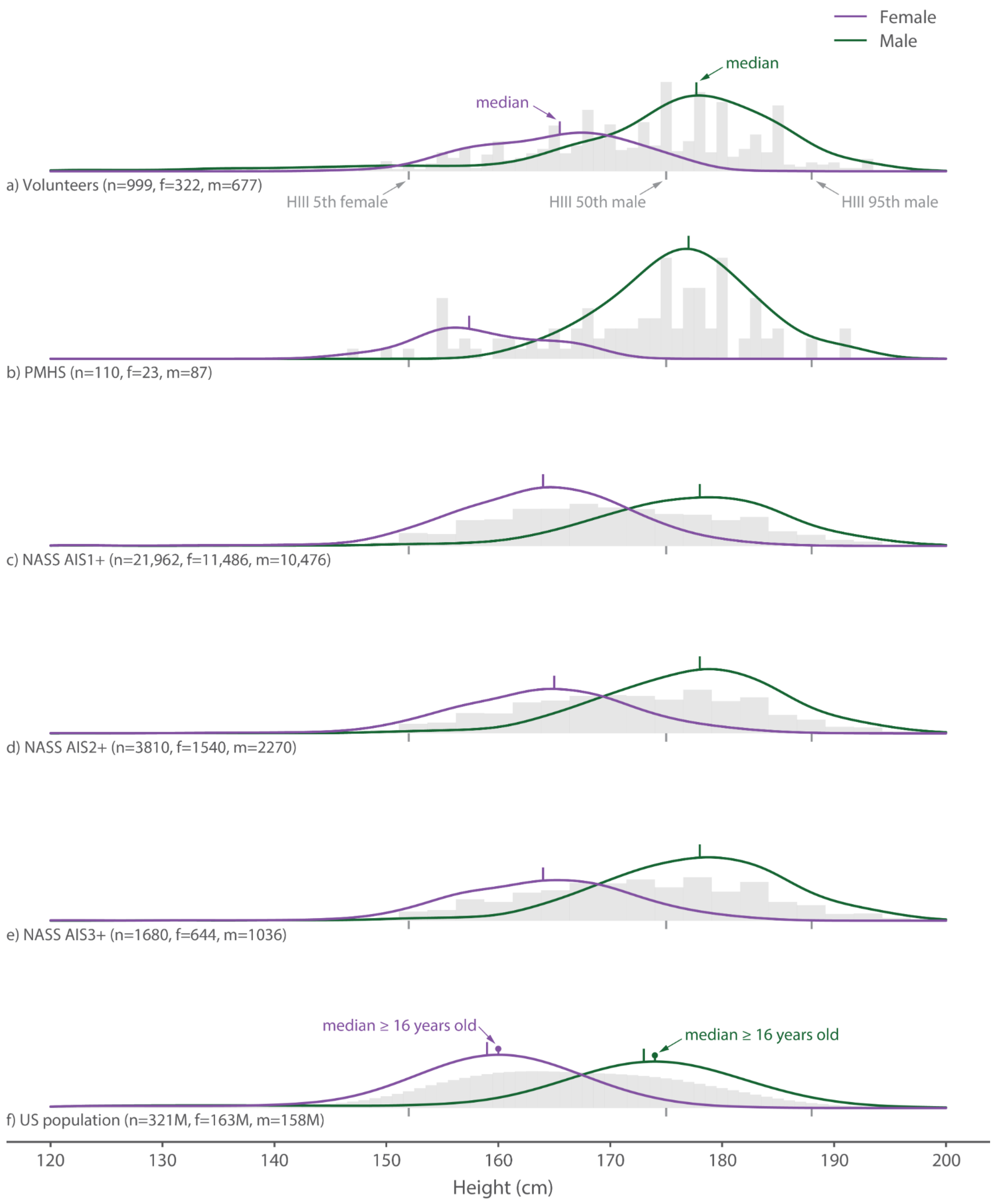

Figure 2. Distribution of all height data (gray histograms), females (purple lines) and males (green lines) for a) the unique volunteers, b) the unique PMHSs, c) the NASS AIS1+ data, d) the NASS AIS2+ data, e) the NASS AIS3+ data, and f) the US population. The gray vertical bars below each histogram show the heights of the 5th percentile female, 50th percentile male, and 95th percentile male Hybrid III crash test dummies.

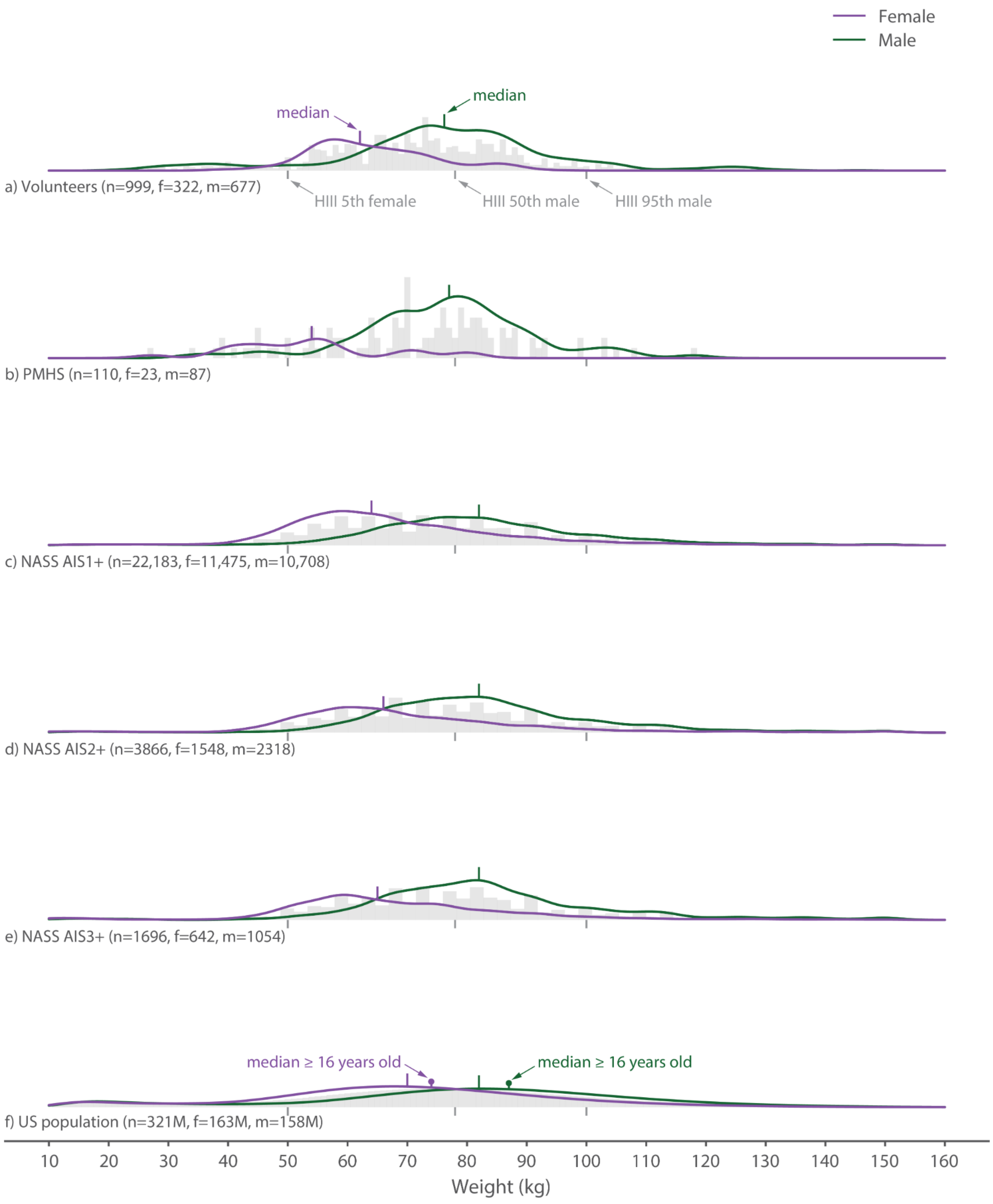

Figure 3. Distribution of all weight data (gray histograms), females (purple lines) and males (green lines) for a) the unique volunteers, b) the unique PMHSs, c) the NASS AIS1+ data, d) the NASS AIS2+ data, e) the NASS AIS3+ data, and f) the US population. The gray vertical bars below each histogram show the weights of the 5th percentile female, 50th percentile male, and 95th percentile male Hybrid III crash test dummies.

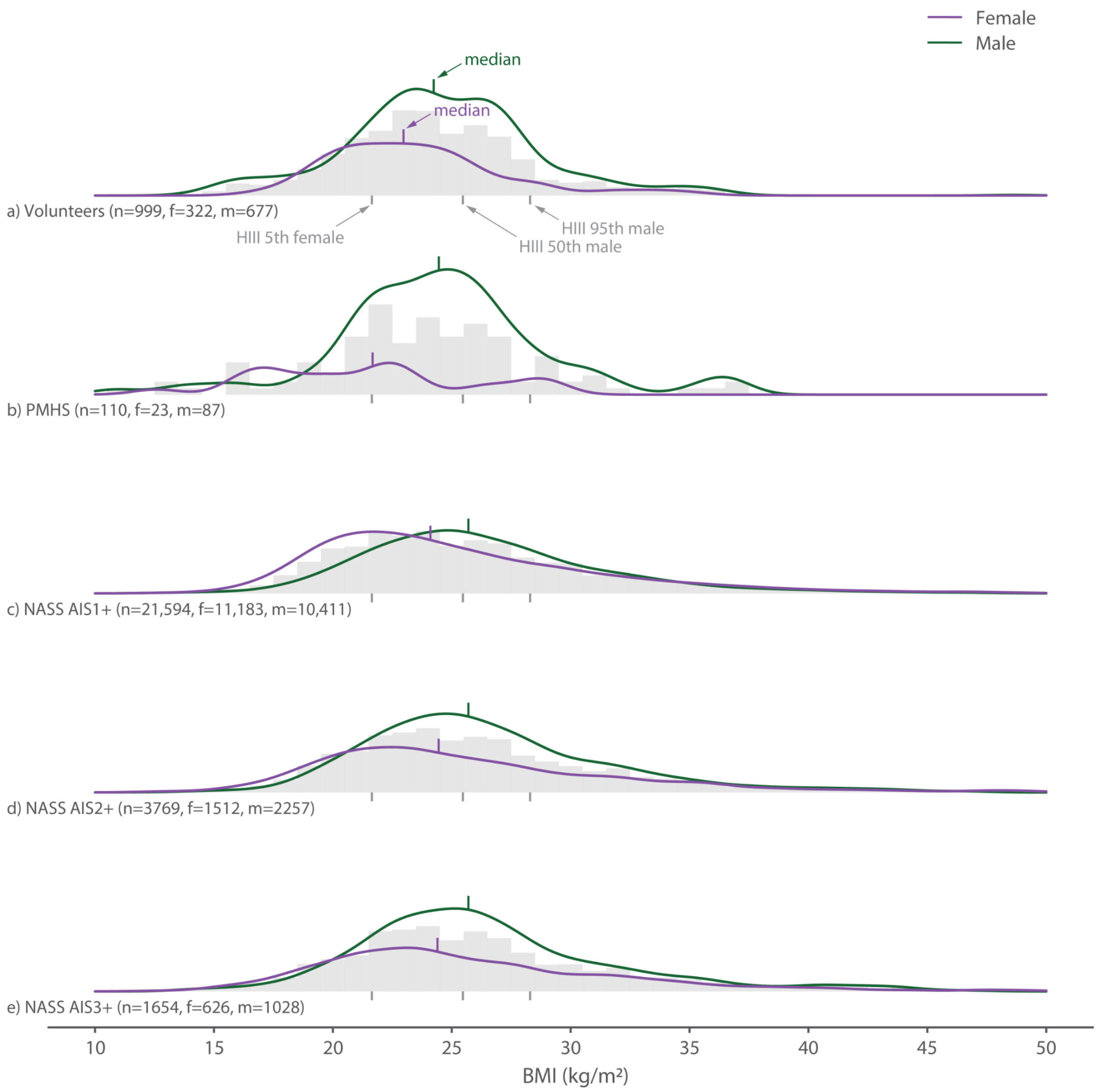

Figure 4. Distribution of all body mass index (BMI) data (gray histograms), females (purple lines) and males (green lines) for a) the unique volunteers, b) the unique PMHSs, c) the NASS AIS1+ data, d) the NASS AIS2+ data, and e) the NASS AIS3+ data. The gray vertical bars below each histogram show the heights of the 5th percentile female, 50th percentile male and 95th percentile male Hybrid III crash test dummies.
